# The targeted overexpression of *SlCDF4* in the fruit enhances tomato size and yield involving gibberellin signalling

**DOI:** 10.1038/s41598-020-67537-x

**Published:** 2020-06-30

**Authors:** Begoña Renau-Morata, Laura Carrillo, Jaime Cebolla-Cornejo, Rosa V. Molina, Raúl Martí, José Domínguez-Figueroa, Jesús Vicente-Carbajosa, Joaquín Medina, Sergio G. Nebauer

**Affiliations:** 1 Plant Physiology Area, Department of Plant Production, Universitat Politècnica de València, Valencia, Spain; 2 Unidad Mixta de Investigación Mejora de la Calidad Agroalimentaria UJI-UPV, COMAV, Universitat Politècnica de València, Valencia, Spain; 3 Centro de Biotecnología y Genómica de Plantas, INIA-Universidad Politécnica de Madrid, Madrid, Spain

**Keywords:** Biotechnology, Plant biotechnology, Plant hormones, Plant molecular biology, Plant physiology, Plant sciences, Plant biotechnology, Agricultural genetics, Molecular engineering in plants, Molecular biology, Transcriptional regulatory elements

## Abstract

Tomato is one of the most widely cultivated vegetable crops and a model for studying fruit biology. Although several genes involved in the traits of fruit quality, development and size have been identified, little is known about the regulatory genes controlling its growth. In this study, we characterized the role of the tomato *SlCDF4* gene in fruit development, a cycling DOF-type transcription factor highly expressed in fruits. The targeted overexpression of *SlCDF4* gene in the fruit induced an increased yield based on a higher amount of both water and dry matter accumulated in the fruits. Accordingly*,* transcript levels of genes involved in water transport and cell division and expansion during the fruit enlargement phase also increased. Furthermore, the larger amount of biomass partitioned to the fruit relied on the greater sink strength of the fruits induced by the increased activity of sucrose-metabolising enzymes. Additionally, our results suggest a positive role of SlCDF4 in the gibberellin-signalling pathway through the modulation of GA_4_ biosynthesis. Finally, the overexpression of *SlCDF4* also promoted changes in the profile of carbon and nitrogen compounds related to fruit quality. Overall, our results unveil SlCDF4 as a new key factor controlling tomato size and composition.

## Introduction

Globally, tomatoes (*Solanum lycopersicum* L.) are one of the most widely cultivated vegetable crops^1^ and play an important role in human nutrition as a rich source of lycopene, minerals and vitamins^2^. During the last century, tomato breeders have mainly focused their efforts on increasing productivity. Nevertheless, additional goals, like fruit shelf-life, taste and quality, or biotic and abiotic resistances, have been progressively addressed^3,4^. Nowadays, flavor has become important, as it is a source of consumer complaint^5^. Therefore, many breeding goals of modern tomato cultivars focus on characteristics that ensure a reliably high yield of high-quality fruits under sustainable conditions.

The final dimensions and weight of the fruits are regulated during multiple stages throughout the development. Cell division is the main process by which fruits increase in size in the early stages. Nevertheless, the cell enlargement stage is considered to be the one that has the greatest impact on fruit size^6^. The spectacular cell expansion that occurs involves importing a great amount of water and sucrose and the accumulation of starch^7^. Endoreduplication events have also been proposed as factors contributing to the increase in fruit size during ontogeny^8^.

Besides the fruit-level processes involved in the determination of the final size, total yield is also influenced by environmental factors, fruit load and, consequently, by photoassimilate availability at whole plant level^7^. Nevertheless, the understanding of processes that influence harvest index is far from complete^9^. The availability of carbon compounds depends on the photosynthetic activity of the source leaves and the partition and allocation of carbon compounds in the plant. Photosynthetically active mature leaves, export fixed C primarily in the form of sucrose, to the sink tissues. Although photosynthesis and sink utilization of carbohydrates are tightly coordinated, a sink-dependent regulation of photosynthesis in leaves has been proposed in tomato^10^. Whilst much is known about the processes that determine photosynthetic efficiency, there is no clear picture about the regulation of processes governing transport and assimilation of photoassimilates into the developing fruit^9^. Fruits are the main sinks in tomato during the reproductive phase^11^, and the amount of sugars accumulated in the fruits is not only dependent on endogenous metabolic processes but also on the degree of phloem unloading. Sucrose-metabolism enzymes, such as invertase, in the fruits maintain the gradient of transport from sources to sinks, and hence, the import into the fruit^11,12^.

Metabolism is a key process for improving fruit production, and several traits, including flavour, nutritional values and health benefits, as well as stress resistance during growth, are affected by the composition of metabolites in fruit tissues. Furthermore, there is an undoubted connection between fruit development and metabolism^13,14^.

Hormones also play a significant role in the processes that lead to mature fruit^15^. Following pollination, fruit set in tomato is achieved through an activation of cell division mainly via the action of auxin and gibberellins^16^. Auxin appears to act partly through gibberellins in the development of the fruit in this species^17^. Fruit growth is likely regulated in the period of cell expansion and endoreduplication by hormones similar to those in fruit set^18^. Gibberellins increase the sink strength of the fruits in several species by the enhancement of phloem unloading or/and the metabolism of carbon assimilates in the fruit^19,20^.

The elaborate physiological and biochemical processes occurring during fruit growth and development require the interplay of numerous gene regulatory networks that are associated with hormonal control mechanisms during ontogeny^21^. Although a great deal of effort has been made to identify the genes involved in the determination of key fruit traits related to quality, development and size^6,13,21–25^, less information is available on the regulatory genes controlling the diverse processes occurring during cell expansion growth phase. Transcription factors (TFs) involved in the control of genes related to fruit patterning and early fruit development, but mainly those connected with fruit ripening and quality, have been reported^21,26–29^. Members of the NAC, GRAS, ERF, TCP, WRKY, MYB, TALE, HD-ZIP and DOF transcription factor families have been proposed after transcriptomic analyses as candidates with putative functions in early fruit development (1–15 days DAA), although functional analyses are lacking^24,29^.

Our group previously reported the CDF1-5 (Cycling DOF Factor) transcription factor gene family in tomato^30^. One of them, *SlCDF4* is mainly expressed in immature green and mature red stages, which might suggest important roles in the regulation of fruit development. In addition, *SlCDF3*, another tomato DOF factor from the CDF group, has been involved, among other processes, in the control of growth, C and N metabolism and fruit production in tomato^31^. These results suggest a key role of members of this family of transcription factors in the regulation of the physiological processes determining growth during the vegetative and reproductive stages of plants.

To explore the impact of SlCDF4 on fruit development, tomato plants overexpressing the *SlCDF4* gene specifically in the fruit were generated. We show that *SlCDF4* overexpression increases fruit growth, regulating the genes involved in cell cycle control, cell growth, water uptake, sink strength and primary metabolism. A higher biomass partition to the fruits is induced and leads to increased yield. In addition, we provide evidence that part of the regulation by *SlCDF4* may be performed by changes in the levels of gibberellin GA_4_ and auxin during fruit development. This study expands our understanding of the functions of *SlCDF4* during tomato fruit development and provides new insights into the regulation of partition and metabolism in the fruit.

## Results

### The expression pattern of *SlCDF4* suggests a role in the regulation of fruit growth

**Figure 1:**
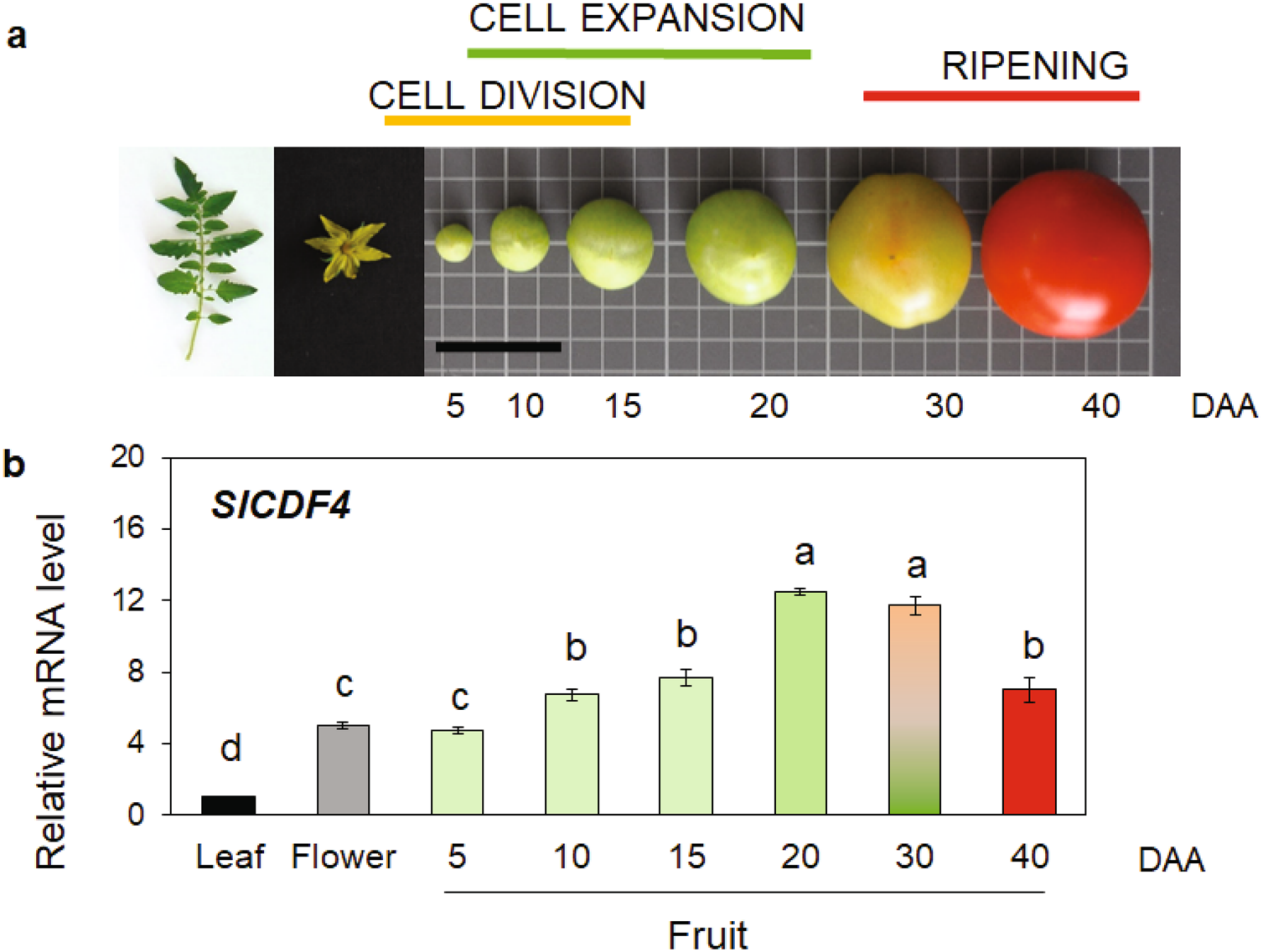
Transcription analysis of *SlCDF4* in non-transformed Moneymaker (MM) tomato fruits during ontogeny. (**a**) Representative examples of the tomatoes analysed in different development stages (*DAA* days after anthesis): 5, 10, 15, 20, 30 and 40 DAA. The cell division and expansion phases of fruit growth are displayed. Bars = 4 cm. (**b**) *SlCDF4* gene transcript levels were analysed by qRT-PCR in tomato fruit in the different stages. Transcript levels were normalized to the values of leaves. Values are mean ± s.e.m. from three biological replicates. Different letters indicate significant differences (LSD test; P < 0.05).

In a previous study we identified the *SlCDF4* gene in tomato, which showed high transcript levels in reproductive organs, mainly in green and red tomato fruits^30^. To explore the function of the gene in greater detail, we studied the expression of the *SlCDF4* gene during fruit development and qRT-PCR analysis was performed in non-transformed Moneymaker (MM) fruits at 5, 10, 15, 20, 30 and 40 DAA (Fig. 1). The results showed that *SlCDF4* mRNA levels increased during the enlargement phase, from 5 to 15 DAA, and were highest at 20–30 DDA (Fig. 1b). Transcript levels of *SlCDF4* in the mature red stage were reduced by 50% when compared to the ones at 20–30 DAA. These results point to a role of SlCDF4 in the regulation of the events occurring during the expansion growth phase.

### The targeted overexpression of the *SlCDF4* in the fruit increases tomato fruit size leading to higher plant yield

In order to gain insight into the physiological function of this gene, we generated tomato lines overexpressing the *SlCDF4* gene specifically in the fruits by the control of the *PEP carboxylase PPC2* promoter (*PPC2::SlCDF4* plants). This promoter has been reported to drive transgene expression during the cell expansion stage of tomato fruit (10 to 35 DDA)^32^. Two homozygous lines (L2 and L6) were selected from the obtained T2 generation.

**Figure 2:**
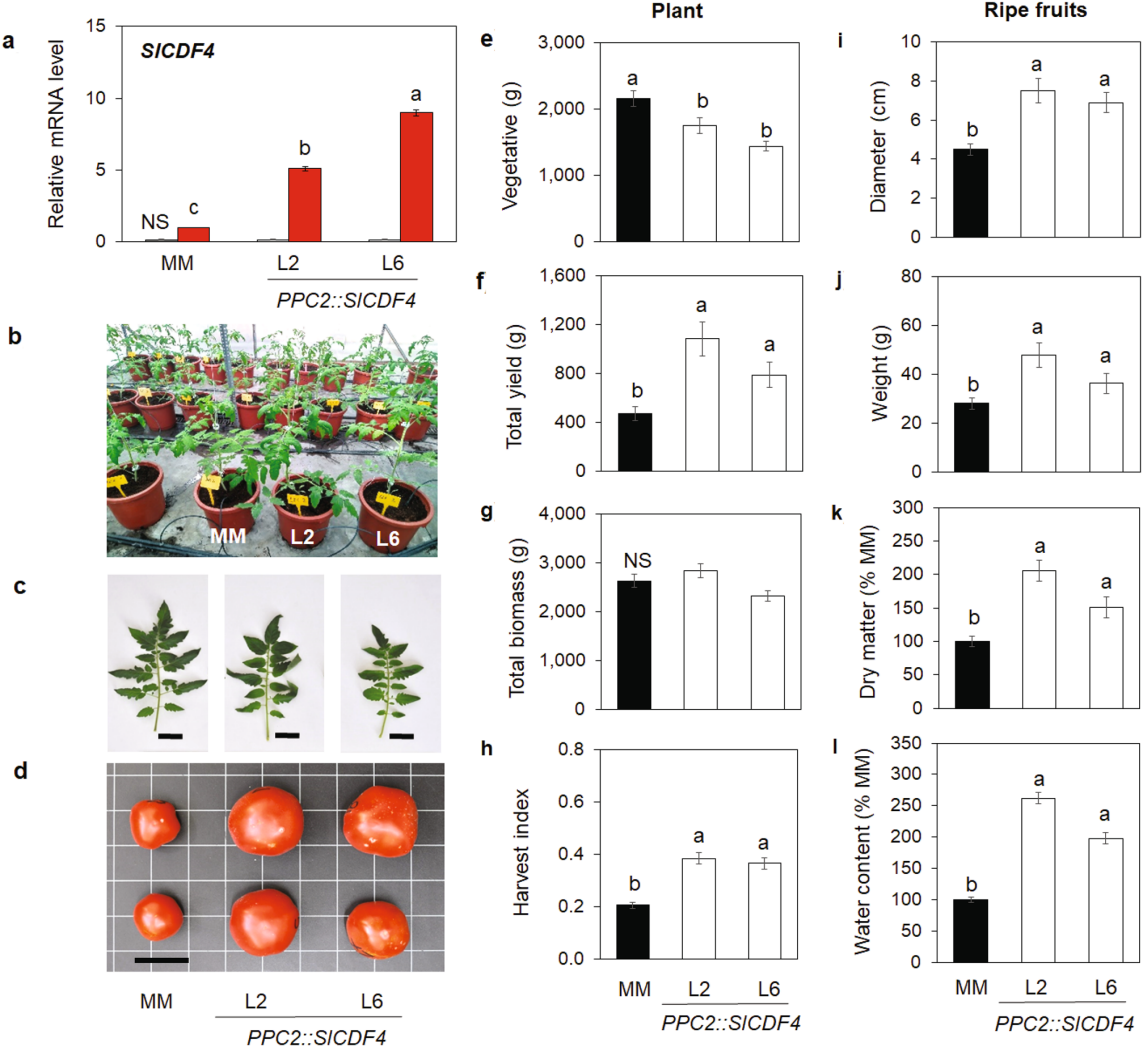
Phenotypic characterization of the MM tomato lines overexpressing the *SlCDF4* gene under the control of the fruit specific *PPC2* promoter. (**a**) Transcript levels of *SlCDF4* in 17 DAA fruits (red bars) and mature leaves (grey bars) of two homozygotic lines for the gene (lines L2 and L6) analyzed by qRT-PCR. Transcript levels were normalized to the values of MM fruit. Representative examples of (**b**) 2-months old plants, (**c**) leaves (4th leaf from the top of a 5-months-old plant at the reproductive stage) and (**d**) red stage tomatoes. Bars = 4 cm. Vegetative biomass (**e**), yield (**f**), total biomass (**g**) and harvest index (**h**) of the *PPC2::SlCDF4* plants at the end of the experiment. Equatorial diameter (**i**) and weight (**j**) in mature red fruits. Dry matter (**k**) and water contents (**l**) in the ripe fruits (% of control). Non-transformed Moneymaker plants (MM) were used as controls. Different letters indicate significant differences (LSD test; P < 0.05). *NS* not significant.

No significant changes in the *SlCDF4* mRNA levels in leaves of *PP2C::SlCDF4* plants were observed when compared to the MM plants (Fig. 2a). In addition, apparent phenotypic differences were not observed during the vegetative phase of growth of the plant (Fig. 2b). Similar number of leaves and plant height were observed in 2-months old plants (Supplemental Fig. S1). In addition, the leaves formed during the vegetative phase also showed similar area (Supplemental Fig. S1). However, the overexpression lines showed slightly smaller leaves during the reproductive phase (Fig. 2c). In accordance, the reduction in vegetative growth resulted in lower biomass of shoots at the end of the experiment (Fig. 2e).

These transgenic lines showed significantly higher transcript levels of the *SlCDF4* gene in the fruits at the expansion phase (17 DAA) than the ones in the MM plants (Fig. 2a). We investigated the impact of the overexpression of *SlCDF4* on the growth and development of the fruits. We found that MM and transgenic plants displayed similar fruit set rates and no apparent differences were observed in the development and ripening of the fruits (Supplemental Fig. S2). The seeds also exhibited similar size and vigor among genotypes (Supplemental Table S1). Nevertheless, the overexpression of *SlCDF4* led to the development of bigger fruits at maturity (up to 160% on average of L2 and L6 lines, compared to MM fruit diameter; Fig. 2d,i,j; Supplementary Table S1). However, no differences in total fruit number were observed. The increased size of the ripe fruits of *PPC2::SlCDF4* plants was related to higher amounts of dry matter per fruit (average of 185% of MM fruits; Fig. 2k) and to a higher water content (average of 235% of MM fruits; Fig. 2l). Furthermore, the bigger fruit size in the *PPC2::SlCDF4* plants led to an improved tomato plant yield (up to 200% on average compared to MM plants; Fig. 2f) and harvest index (Fig. 2h). Together, our data suggest that *SlCDF4* overexpression impacts on the tomato structure and size and, consequently, on fruit production through the regulation of dry matter accumulation and water uptake into the fruits. Since total plant biomass was similar between MM and *PP2C:SlCDF4* plants at the end of the experiment (Fig. 2g), the higher yield observed in the transgenic plants was at the expense of a reduced vegetative biomass.

### *SlCDF4* overexpression in the fruit affects both cell expansion and division during early fruit development

**Figure 3:**
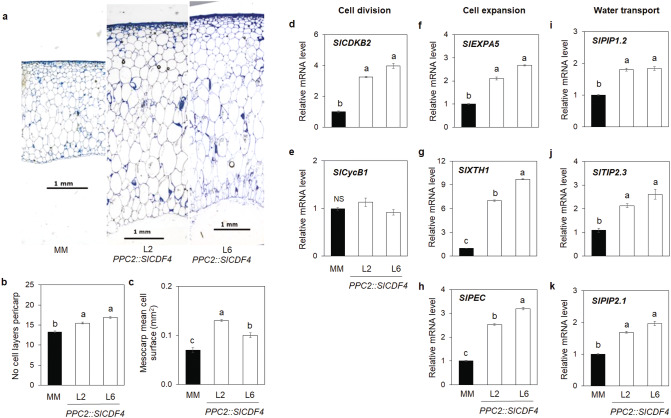
Effect of the overexpression of *SlCDF4* in the fruit on cell size and number in the pericarp of fruits. (**a**) Micrograph of the number of cell layers and cell size at breaker stage. (**b**) Average number of cell layers and (**c**) mean cell area of the pericarp of MM and transgenic lines (L2 and L6) at breaker stage. Data are the mean (± s.e.m.) of ten different fruits of different plants. Bars = 1 mm. The mRNA levels of genes (**d**) *cyclin dependent kinase SlCDKB2* and (**e**) *cyclin SlCycB1* (Cell division), (**f**) *expansin SlEXPA5*, (**g**) *xyloglucan endotransglycosilase SlXTH1* and (**h**) *pectate lyase SlPEC* (Cell expansion) and *aquaporins* (**i**) *SlPIP1.2*, (**j**) *SlTIP2.3* and (**k**) *SlPIP2.1* (Water uptake) were determined in fruits at the expansion phase (17 DAA). Data are the mean (± s.e.m.) of three biological replicates. Non transformed plants (MM) were used as controls. Transcript levels were normalized to the values of MM. Different letters indicate significant differences (LSD test; P < 0.05). *NS* not significant. The micrographs were captured with a Nikon DS-Fi3 camera using the Nikon NIS element D software (http://www.nikon.com/products. Nikon, USA).

Tomato fruit size is determined by both the cell divisions occurring during the first 8 to 12 DAA, and by cell expansion lasting from 8 to 30 DAA^6^ (Fig. 1a). To understand the processes explaining the increased size of the *PPC2::SlCDF4* fruits, histological cross sections of the pericarp tissue were analysed after the end of the expansion growth phase, at breaker stage. In this stage, the transgenic fruits had a significantly thicker pericarp (5.3 mm on average vs. 2.9 mm of MM fruits; Fig. 3a and Supplementary Table S1). Accordingly, an increase in the number of cell layers in the pericarp, including exo- and mesocarp, were observed in the fruits of the *PPC2:SlCDF4* lines when compared to MM (15.5 on average vs. 13.3, respectively; Fig. 3b and Supplementary Table S1). Moreover, the mean cell size of the mesocarp was larger in the transgenic lines than in MM plants (0.11 mm^2^ on average vs. 0.07 mm^2^, respectively; Fig. 3c and Supplementary Table S1). Together, the increased number of cell layers and cell area explained the larger size of the ripe fruits of the *PPC2::SlCDF4* plants.

To gain insight into the molecular mechanisms underlying the increased fruit size of *PPC2::SlCDF4* plants, we performed qRT-PCR analyses in fruits of the different marker genes involved in cell cycle control, like the B2-type cyclin dependent kinase *SlCDKB2* and the cyclin *SlCyclinB1*, cell wall growth, like the expansin *SlEXPA5*, xyloglucan endotransglycoxylase-hydrolase *SlXTH1* and pectate lyase *SlPEC17*, and water uptake, like the tonoplast intrinsic *SlTIP2.3* and the plasma membrane intrinsic *SlPIP1.2* and *SlPIP2.1* aquaporins (Fig. 3)^16^. Analyses were performed by the middle of the expansion growth period (17 DAA) since these genes display higher mRNA levels between 5 and 20 DAA in tomato fruit (Supplementary Fig. S3).

As shown in Fig. 3d, the overexpression of *CDF4* up-regulates the *SlCDKB2* gene, previously related to increased cell division rates in fruits^16^. Furthermore, the transcript levels of *SlEXPA5*, *SlXTH1* and *SlPEC17* involved in cell growth in tomatoes were also higher in the *PPC2::SlCDF4* plants (Fig. 3f–h). Consistently, increased cell wall content was found in the transgenic fruits at maturity (Supplemental Table S1). Furthermore, higher transcript levels of *SlTIP2.3*, *SlPIP1.2* and *SlPIP2.1 aquaporins* were observed (Fig. 3i–k). These results suggest that the targeted *SlCDF4* overexpression in fruits during the expansion phase promotes significant changes in the expression of genes related to cell wall growth and water uptake by the cell to sustain the increase in cell volume.

Endoreduplication is a remarkable characteristic of the fleshy pericarp tissue of developing tomato fruits, and has been proposed as a morphogenetic factor acting in support of cell growth^33^. In order to assess whether this process also contributes to the increased fruit growth of *PPC2::SlCDF4* plants, we determined the ploidy of the pericarp of fruits at breaker stage. Our results showed that the fruits of both genotypes displayed a similar range and frequency of ploidy levels, indicating no differences in the endoreduplication events (Supplementary Fig. S4). We also analysed by qRT-PCR the transcript levels of the cyclin-dependent kinase inhibitor *SlKRP1*, the cell cycle-associated protein kinase *SlWEE1* and the anaphase-promoter complex activator *SlCCS52A* genes, involved in endocycle control during cell expansion, in 17 DAA fruits (Supplemental Fig. S4)^23^. All three genes showed similar mRNA levels among the studied genotypes, in accordance with the observed ploidy levels.

Taken together, these results suggest that *SlCDF4* might be involved in fruit size determination through the regulation of target genes promoting cell divisions and enlargement, but independently of endoreduplication. Interestingly, several DOF binding sites were found in the promoters of the studied genes (Supplementary Fig. S5), indicating that they might be direct targets of the CDF4 TF.

### *SlCDF4* induces the accumulation of gibberellin GA_4_ content in fruits during the cell expansion phase

**Figure 4:**
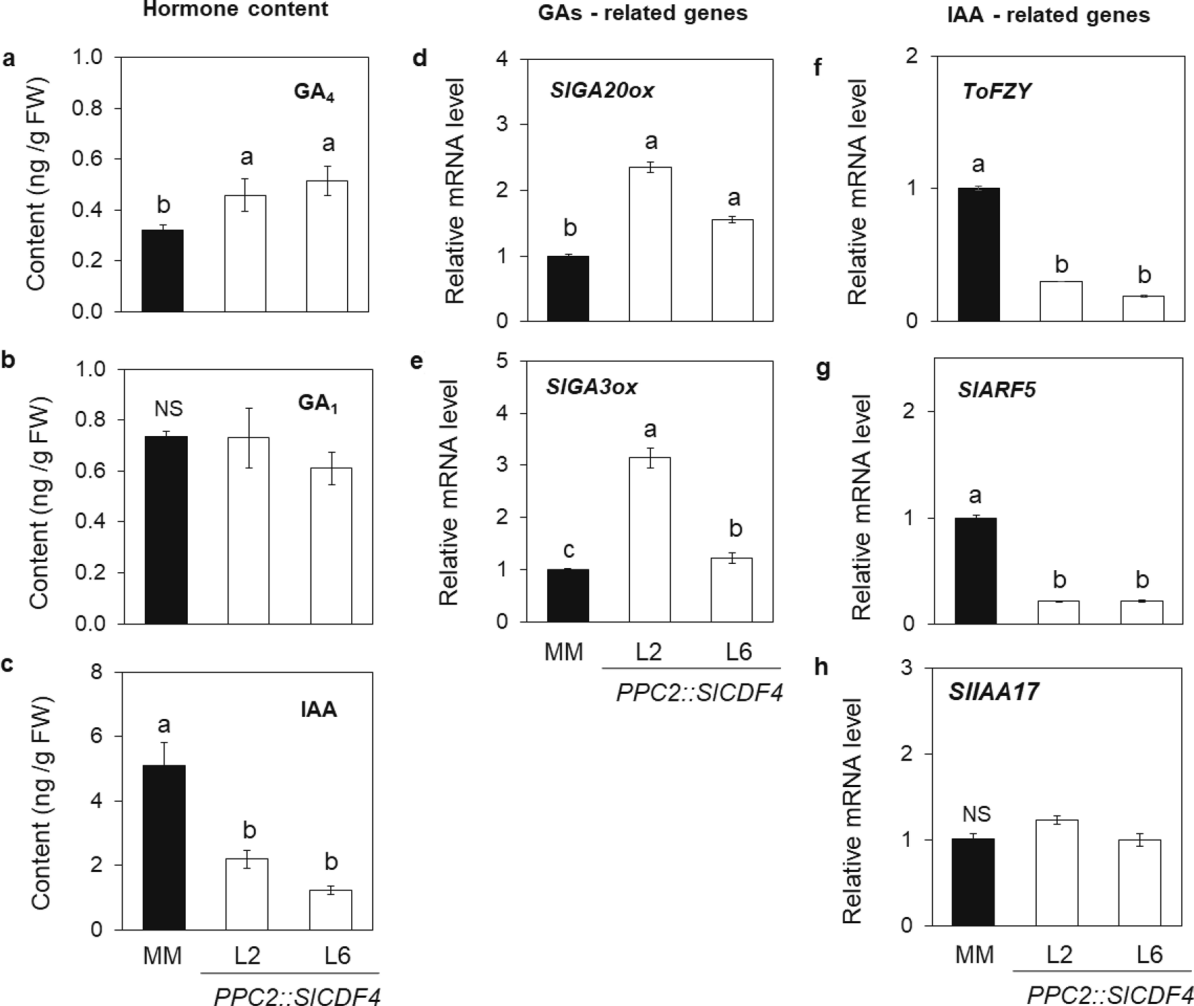
Gibberellin and auxin biosynthesis in *PPC2::SlCDF4* fruits. Gibberellins GA_4_ (**a**), GA_1_ (**b**) and auxin IAA (**c**) contents were determined in the expansion phase of fruit growth (17 DAA) in the transgenic lines (L2 and L6). Fruits of non-transformed plants (MM) were used as controls. The transcript levels of the gibberellin-related *GA20 oxidase GA20ox* (**d**) and *GA3 oxidase GA3ox* (**e**) genes, and the auxin-related *flavin monoxygenase ToFZY* (**f**), auxin response factor 5 *SlARF5* (**g**) and Aux/IAA transcription factor *SlIAA17* (**h**) genes were determined by qRT-PCR in 17 DAA fruits. Data are the mean (± s.e.m.) of three biological replicates. Non-transformed plants (MM) were used as controls. Transcript levels were normalized to the values of MM. Different letters indicate significant differences (LSD test; P < 0.05). *NS* not significant.

Auxins, but mainly gibberellins, are known to regulate cell cycle and cell expansion genes, and determine the final fruit size in tomato^34–36^. To further investigate the physiological role of SlCDF4, we determined the content of the bioactive gibberellins GA_1_ and GA_4_, and indole-acetic acid (IAA) in green fruits (17 DAA) of *PPC2::SlCDF4* and MM plants. As shown in Fig. 4, GA_4_ levels increased in the *PPC2::SlCDF4* lines when compared to MM plants. However, no differences were observed in the GA_1_ content of genotypes.

In order to assess whether SlCDF4 might directly regulate the biosynthesis of GA_4_, we analysed the mRNA contents of the *SlGA3ox1* and *SlGA20ox1* biosynthetic genes in 17 DAA fruits by qRT-PCR^35^. The *PPC2::SlCDF4* plants showed increased transcript levels of both the *GA3 oxidase SlGA3ox1* and *GA20 oxidase SlGA20ox1* genes (Fig. 4d,e), suggesting that the observed effects of *CDF4* on fruit growth are mediated, at least partly, by gibberellins. Additionally, we observed lower auxin levels in the *PPC2::SlCDF4* fruits (17 DAA) (Fig. 4c). Accordingly, the transcript levels of *ToFZY* flavin monoxygenase, a YUCCA-like gene involved in auxin biosynthesis^37^, were higher in the 17 DAA MM fruits (Fig. 4f). To further understand the relationship of SlCDF4 with the auxin and gibberellin signalling pathways, we also investigated the expression of the AUXIN RESPONSE FACTOR 5 (*SlARF5*) gene, involved in the determination of tomato fruit size, and the Aux/IAA transcription factor *SlIAA17*, reported to participate in auxin signalling during the fruit cell expansion^16,23^. Interestingly, we observed lower *SlARF5* transcripts in the *PPC2::SlCDF4* fruits than the ones in control plants (Fig. 4g), but no differences were observed in *SlIAA17* mRNA levels (Fig. 4h).

These results suggest that CDF4 might play a complex dual role, controlling the biosynthesis of both gibberellins and auxins during fruit development and regulating, at least partly, the ToFZY-ARF5 module. CDF4 might exert functions through either an indirect effect on GA and auxin related genes by the regulation of ARF5 or by a direct effect over GA/auxin related target genes. As a result, GA_4_ is accumulated in the growing green fruits, and this may lead to the increased cell number and cell growth, culminating in the development of larger fruits.

### An increase in fruit sink strength drives the higher fruit yield in the *PPC2::SlCDF4* plants

**Figure 5:**
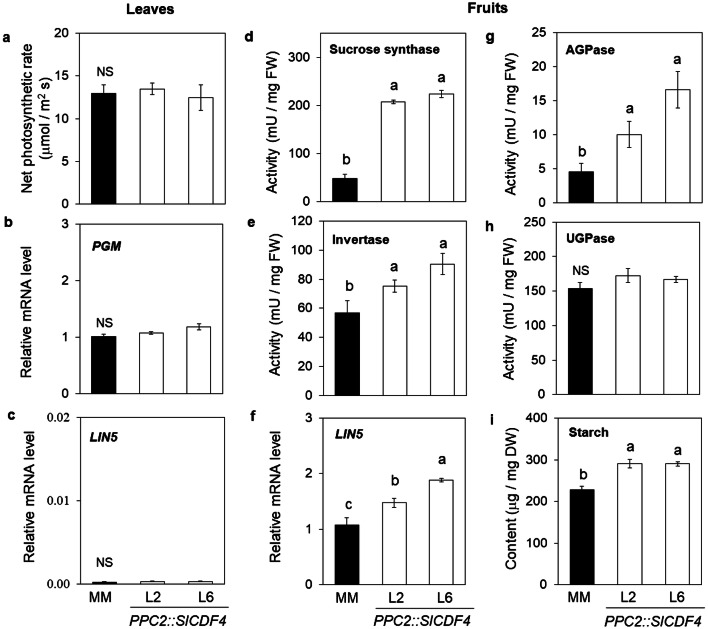
Changes in biomass partition at plant level by the expression of *SlCDF4* in the fruits. Net photosynthetic rate (**a**), and (**b**) *phosphoglucomutase* (*PGM*) and (**c**) *acid invertase* (*LIN5*) transcripts levels were determined in mature leaves. Photosynthesis values are mean (± s.e.m.) of determinations in ten different plants. The effect of the overexpression of *SlCDF4* (lines L2 and L6) in the fruit on enzyme activities related to carbohydrate utilization and storage was assessed. Sucrose synthase (**d**), acid invertase (**e**), ADP-glucose pyrophosphorylase (AGPase) (**g**) and UDP-glucose pyrophosphorylase (UGPase) (**h**) activities were determined in 17 DAA fruits. Transcript levels of *cell wall invertase LIN5* (**f**) and starch content (**i**) were analyzed in the same fruits. Enzymatic, metabolic and transcriptomic data are the mean (± s.e.m.) of three biological replicates. Non transformed plants (MM) were used as controls. Transcript levels were normalized to the values of MM. *LIN5* leaf mRNA levels were normalized to the values of MM fruit. Different letters indicate significant differences (LSD test; P < 0.05). *NS* not significant.

To investigate the underlying physiological mechanisms supporting the increased fruit growth and higher yield of the *PPC2::SlCDF4* plants, the net photosynthetic rate and biomass partition in the plants were determined. *PPC2::SlCDF4* and MM plants displayed similar net photosynthetic rates (Fig. 5a), indicating that no changes were induced among genotypes in the net biomass gain at plant level. This result might support that the similar total biomass per plant described for *PPC2::SlCDF4* and MM lines at the end of the experiment (Fig. 2g). Moreover, similar *cytosolic phosphoglucomutase* (*PGM*) transcript levels were found by qRT-PCR in mature leaves (Fig. 5b), indicating that no changes were induced in the carbon allocation of source leaves in *PP2C::SlCDF4* plants.

Thus, the overexpression of *SlCDF4* in the tomato fruit provoked a higher photoassimilate partition to the fruits, whereas less biomass was accumulated in their vegetative organs (Fig. 2e,f). Sink activity in tomato has been closely related with the activity of the enzymes involved in carbohydrate utilization and storage^38,39^. To assess whether the greater sink strength of the tomato fruits overexpressing the *SlCDF4* gene is controlled by enzymes related to the metabolism of the phloem-imported sucrose, the activities of invertase and sucrose synthase have been determined in young green fruits (17 DAA) during the expansion phase (Fig. 5d,e). Additionally, AGP-glucose and UGP-glucose pyrophosphorylase (AGPase and UGPase) activities, related to the allocation of carbon resources for growth and storage, respectively, have also been measured (Fig. 5g,h).

Notably, a marked increase (fourfold) in sucrose synthase activity was observed in the fruits of *PPC2::SlCDF4* plants (Fig. 5d). Invertase activity was also greater in the fruits overexpressing the *SlCDF4* gene (Fig. 5e). The increased invertase activity correlated with higher transcript levels of the cell wall invertase gene *LIN5* (Fig. 5f). Almost no *LIN5* transcripts were detected in source leaves (Fig. 5c). The levels of UGPase activity, related to carbohydrate metabolism in sink organs, did not differ greatly from genotype to genotype, whereas AGPase activity, related to starch synthesis increased in both *PPC2::SlCDF4* lines compared to MM fruits (Fig. 5g,h). Accordingly, starch content was higher in the transgenic green fruits (290 μg/mg DW starch on average vs. 228 μg/mg DW in MM plants; Fig. 5i).

Taken together, our data suggest that the overexpression of *SlCDF4* in tomato fruits increases the partition of carbon resources to the fruits by increasing the sink strength through changes in the activity of sucrose-metabolising, mainly sucrose synthase and starch synthesising enzymes.

### The overexpression of *SlCDF4* in the fruit leads to changes in the composition of tomato quality-related compounds

**Figure 6:**
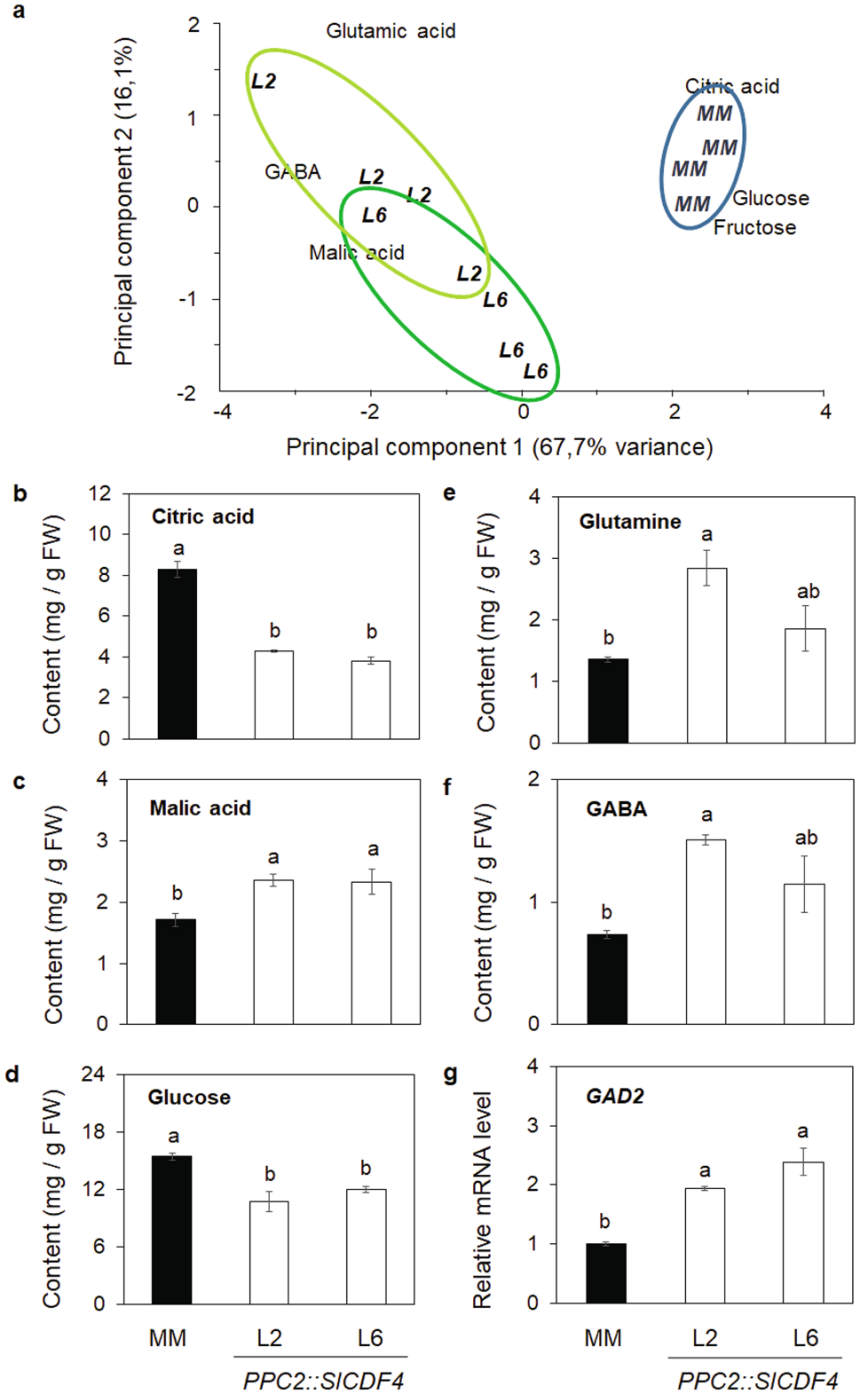
Effect of the overexpression of *SlCDF4* in the fruit on compounds related to fruit quality at the mature red stage. (**a**) PCA analysis for the comparison of ripe fruit metabolic profiles related to fruit quality in *PPC2::SlCDF4* plants (lines L2 and L6). Citric acid (**b**), malic acid (**c**), glucose (**d**), glutamine (**e**) and GABA (**f**) contents in mature red fruits. The mRNA levels of *glutamate decarboxylase* (*GAD2*) gene (**g**) were determined in fruits at the expansion phase (17 DAA). Transcript levels were normalized to the values of MM. Data are the mean (± s.e.m.) of four biological replicates. Non-transformed plants (MM) were used as controls. Different letters indicate significant differences (LSD test; P < 0.05). *NS* not significant.

To investigate whether the overexpression of *SlCDF4* also impacts on the metabolic profile of the carbon compounds related to fruit quality, the accumulation of organic acids and soluble sugars was determined in mature fruits. A PCA analysis reveals that the *PPC2::SlCDF4* lines group in different plots than the MM fruits (Fig. 6a). Among the compounds quantified, a higher malic acid content and lower citric acid and glucose contents were observed in the *PPC2::SlCDF4* fruits (Fig. 6b–d; Supplementary Table S2). Accordingly, lower sugar content of the transgenic fruits was related to lower Brix degrees. These changes led to an increased malic acid to citric acid ratio and higher sucrose equivalents to citric acid ratio, both parameters related to sensorial perception (Supplementary Table S2).

It has to be noted that *PPC2::SlCDF4* fruits had a higher GABA and glutamine content (Fig. 6e and Supplementary Table S2). Accordingly, the transgenic fruits displayed higher *glutamate decarboxylase* (*GAD2*) transcript levels (average of 216% of MM fruits in L2 and L6 lines; Fig. 6f).

Overall, these data indicate that the overexpression of *SlCDF4* leads to changes in the composition of the carbon compounds related to fruit quality.

## Discussion

Fruit size and quality traits are fundamentally determined by processes occurring during the cell expansion phase in the early stage of tomato fruit development^14^. Fruit growth in this phase is sustained by a large increase in cell volume linked to membrane and cell wall synthesis, and the accumulation of water, mineral ions and metabolites. Several transcription factors participate in the complex regulatory networks controlling the fruit development^14,21,29,40^.

In this study, we suggested SlCDF4 as a novel transcription factor involved in tomato fruit growth. We showed that the CDF4 is highly expressed during the green phase of growth of tomato fruit development. Our results indicate that the targeted overexpression of *SlCDF4* in the fruit activates the expression of genes involved in cell expansion and division. In addition, SlCDF4 also influences the amount of photoassimilates partitioned to the fruits. As result, we reported that a significant increase in fruit size and plant yield is induced when overexpressed specifically in the fruits.

Tomato fruit size is determined by cell division, cell expansion and endoreduplication processes occurring during its development^7,41^. Classically, there has been proposed a correlation between fruit size and cell number, yet cell expansion may contribute up to 90% of the increase in fruit weight^24^. In the present study, we performed a molecular characterization analysis to identify putative target genes of the *SlCDF4* in the fruit and determine the cell processes explaining the larger fruit size observed in the *PPC2::SlCDF4* plants. The overexpression of *SlCDF4* increased both cell expansion- and cell division-related genes in tomato fruits during the cell expansion phase. These results indicate that *SlCDF4* might play a role in both processes determining fruit size. The increased number of cell layers in the fruit pericarp of *PPC2::SlCDF4* plants was related to the higher mRNA levels of *SlCDKB2 cyclin-dependent kinase*. Cell expansion genes, *SlEXPA5, SlXTH1* and *SlPEC17,* were also up-regulated. The increase in *expansin* and *XTH* mRNAs was also described in plants overexpressing the *CDF3* gene in tomato^31^. These data reinforce the role of CDF genes in growth control during plant development. Furthermore, the expression of several *aquaporin* genes was also up-regulated, in accordance with the increased water content of *PPC2::SlCDF4* fruits. These observations are consistent with previous studies, which showed that aquaporins are involved in water flux during tomato fruit development and crucial in determining fruit size^42,43^. It has to be pointed out that *SlCDF4* overexpression had no impact on endoreduplication events in the pericarp, thus, ruling the role any involvement of this process in the reported regulation of fruit growth.

Tomato yield reflects the proportion of biomass invested in the fruits as a result of the balance between the activities of sources and sinks. Thus, partitioning is thought to be a major determinant of fruit production^9^. The rates of phloem unloading and assimilate allocation in sinks are key components in this framework, and knowledge of the regulation of the processes involved offers additional targets to enhance yield potentials.

Photosynthesis and sink utilization of carbohydrates under non-limiting conditions are tightly coordinated^10,44^. At the end of the experiment, we reported that *PPC2::SlCDF4* and MM plants had similar carbon fixation rates and total accumulated plant biomass. Since the overexpression of the *SlCDF4* is restricted to the fruits, no differences were expected in the rates of phloem loading between genotypes. Thus, the increased fruit production observed in the transgenic plants was related to changes in photoassimilate partitioning at plant level. To confirm this, we characterized the sink strength of the fruits at molecular level, and the activities of sucrose metabolising enzymes were determined in green fruits during the expansion phase. Sucrose synthase and apoplastic invertase have been involved in the metabolism and uptake of sucrose from the phloem, and thus, in the control of the carbon flux into the fruits during the active growth phase^39,45,46^. We reported a significant increase in invertase and sucrose synthase activities in the fruits of *PPC2::SlCDF4* plants. These results confirmed the relationship of both sucrolytic activities with the sink strength of the fruits. Accordingly, higher *cell wall invertase LIN5* transcript levels were observed in the transgenic fruits. The tomato *LIN5* gene has been described as a ‘sink gene’, controlling sugar import in the fruit pericarp^47^. Similarly, Ikeda et al.^48^ also related *cell wall invertase 6* (LIN6) and *sucrose synthase* (TOMSSF) expression during the expansion phase of the fruit (20 to 30 DAA) with an increased photoassimilate metabolism. The authors suggested that their expression was regulated by a common unknown transcription factor. Our data would suggest that SlCDF4 might be involved in the control of the sink strength of the fruits through the regulation of sucrolytic activities.

Sink activity also includes carbohydrate metabolism and storage, thus maintaining the sucrose gradient and transport between source and sink organs^49^. In the early rapid growth stage, a fruit accumulates imported assimilates, mainly in the form of hexoses and starch. Coherently, fruits of the *PPC2::SlCDF4* plants had greater ADP-glucose pyrophosphorylase (AGPase) activity, the rate-limiting enzyme of the starch synthesis pathway, and starch content. These results concur with the reported correlation between sucrose synthase activity and starch accumulation in tomato fruits^39^. Taken together, our results indicate that overexpression of *SlCDF4* promotes changes in photoassimilate partitioning through the control of the flux and allocation of carbon compounds to the fruit. The greater sink strength of the fruits observed in the *PPC2::SlCDF4* plants supported the increased biomass accumulation per fruit.

The higher dry matter observed in the transgenic fruits at maturity was consistent with the increased number of cells and cell size. In addition to the larger cell wall layer surface, the *PP2C::SlCDF4* fruits also displayed an enriched cell wall content (Supplemental Table S1). Both the increased dry matter together with the increased water content, explained the higher weight of the *PP2C::SlCDF4* fruits.

Primary metabolism is essential for fruit growth and quality^50^. Both the metabolism and the accumulation of carbohydrates, organic acids and amino acids in tomatoes depend on the developmental stage of the fruit. In later development stages, starch breaks down leading to the accumulation of hexoses, along with a decline in organic acids^52,53^. We determined key primary metabolites in mature red fruits to assess whether the overexpression of *CDF4* also impacts on fruit quality.

The overexpression of *SlCDF4* in the fruit induced significant changes in the profile of primary metabolites at maturity. The transgenic fruits accumulated higher malate and GABA contents. These observations concur with previously reported metabolomic analyses of plants overexpressing *CDF3* genes in Arabidopsis and tomato^31,51^. The changes in TCA metabolites and GABA in the *PPC2::SlCDF4* are compatible with an altered GABA shunt regulation. A similar profile has also been observed in the *35S::AtCDF3* tomato plants^31^, thus suggesting that SlCDF genes might be involved in the regulation of anaplerotic pathways of the TCA cycle.

In tomatoes, both cell wall invertase activity and starch accumulation during the expansion phase have been related to sugar content at maturity^47,52,53^. This was not observed in our study, since *PPC2::SlCDF4* fruits showed, besides the slight dilution effect provoked by the higher water content, lower sugar content when compared to MM fruits. As transgenic and control fruits showed similar low starch content at the ripe stage (Supplemental Table S2), the targeted overexpression of *SlCDF4* in the fruit might provoke changes in the allocation of the imported carbon compounds.

Integrated metabolite and transcriptomic analyses in tomato fruit indicate the dominance of a post-translational regulation of its primary metabolism^13^. Nevertheless, changes in the expression levels of several metabolite biosynthesis genes during fruit development have been reported^14^. Furthermore, metabolite contents were related to the expression of different transcription factors participating in the regulatory network hubs. The metabolite profile data of the transgenic fruits presented here suggest that SlCDF4 could also participate in the regulation of the fruit primary metabolism during development and influence fruit quality at maturity. The specific targets of *SlCDF4* in the determination of the fruit metabolism must be further elucidated. Interestingly, the overexpression of *SlCDF4* also promoted higher levels of *SlCDF2* and *SlCDF5* (Fig. S6). These results suggested that the role of SlCDF4 in fruit development might involve, directly or indirectly, other CDFs.

Gibberellins play a crucial role in tomato growth control in early developmental stages^54^. The level of active gibberellins has been related to the control of the fruit size and weight through the activation of genes involved in cell division and expansion^55,56^. These effects are also confirmed by our data, showing higher gibberellin GA_4_ content and an increased number of cell layers and larger cell size in the pericarp of the fruit of *PPC2::SlCDF4* plants.

Furthermore, the reported increase in the sink strength of the transgenic tomatoes coincides with previous studies linking gibberellin levels with a higher capacity of the fruits to import carbon compounds through the activation of sucrose-cleaving enzymes^19^. The results of our study provide functional evidence supporting that SlCDF4 might display functions in the determination of fruit size and sink demand, involving gibberellins. Accordingly, the overexpression of *SlCDF4* induced higher levels of the *GA20ox* and *GA3ox* transcripts that encode for the enzymes of active gibberellin biosynthesis^17^.

In tomato, gibberellins interact with auxins to drive fruit growth and development^57^. Several members of the auxin signalling pathways (ARFs and Aux/IAA) are involved in the control of cell division and expansion during early fruit development^16,58^. This regulation is exerted by auxins together with gibberellins through complex crosstalk pathways. Recently, *SlARF5* has been proposed as a key factor in the control of fruit growth and the regulation of auxin/gibberellin signalling pathways during tomato development^16^. In this study, *SlARF5* suppression reduced the number of pericarp layers and fruit size, and downregulated gibberellin biosynthesis *GA20ox* gene. However, some genes involved in the gibberellin-signalling pathway were up-regulated in the *ami*RNA *SlARF5* lines. We reported that the overexpression of *SlCDF4* in fruits promoted the downregulation of *SlARF5* transcripts but significantly increased fruit size and gibberellin GA_4_ content compared to controls. These results suggest a complex regulatory interaction between both transcription factors in the control of the crosstalk between auxins and gibberellins during tomato fruit development. Further work is needed to determine the specific functions and interplay between these factors.

Our data indicated that SlCDF4 might play important functions in fruit growth and development. The *SlCDF4* gene showed high expression in the fruit during the expansion phase and up-regulated important genes related both to cell expansion and division and water uptake when overexpressed specifically in the fruit. In addition, the fruit of *PPC2::SlCDF4* plants displayed higher photoassimilate partition to the fruits through increased activity cell wall invertase, sucrose synthase and ADP-glucose pyrophosphorylase. Together, these changes led to increased fruit size and total plant yield. The overexpression of *SlCDF4* also impacted on the content of carbon and nitrogen compounds in the mature red fruit, suggesting a role in the regulation of primary metabolism and, as result, in the fruit quality. Finally, we reported that gibberellins play a role in mediating the changes in the development of the fruit, since the biosynthesis of GA_4_ was upregulated in the *PPC2::SlCDF4* fruits in the expansion phase.

## Materials and methods

### Plant material and growing conditions

*Solanum lycopersicum* CDF4 gene (*SlCDF4*) was overexpressed in the tomato fruit under the control of the fruit-specific tomato PEP carboxylase *SlPPC2* promoter^32^. Tomato (*S. lycopersicum*) cv. ‘Moneymaker’ was transformed following the method described by Ellul et al.^59^. Two rounds of selection for kanamycin-resistant seedlings were conducted to determine homozygous lines as described in Renau-Morata et al.^31^. Two independent lines (L2 and L6) harbouring one copy of the construction were characterized in the present study. Non-transformed Moneymaker (MM) tomato plants were used as controls.

Seeds were germinated on a moistened mixture of peat moss and sand in the greenhouse. Seedlings were transferred to 15 L pots that contained coconut coir fibre and were irrigated with Hoagland no. 2 nutrient solution^31^. Plants were cultured for 6 months in a greenhouse covered with a reflective aluminised net. The maximum light (PAR) in the greenhouse was approximately 500 μmol m^−2^ s^−1^, and the temperature ranged between 20 (minimum) and 35 °C (maximum). Ten different plants of each genotype were used in the experiments.

### Photosynthetic activity determinations

The net CO_2_ assimilation rate (A_N_, μmol m^−2^ s^−1^) and related gas exchange parameters were determined in steady state conditions with an LI-6400 infrared gas analyser (LICOR Biosciences, Lincoln, NE, USA)^31^. One measurement per plant was taken on the third or fourth leaf from the apex. Ten different plants were used. The conditions in the measuring chamber were 500 mol s^−1^ of flow rate, a saturating PAR of 1,000 μmol m^−2^ s^−1^, 400 ppm CO_2_ and 60–70% relative humidity.

### Biomass quantification

The fresh and dry weights of shoot and root systems were determined at the end of the experiment^31^. The agronomic performance of the transgenic lines was assessed by measuring total yield (g plant^−1^), number of fruits (no plant^−1^) and fruit weight (g fruit^−1^). Each fruit was harvested at maturity until the 4th truss. Harvest index (HI) was calculated as the ratio between the total yield and total biomass of the plant. No incidence of Blossom-End Rot (BER) was observed among the studied lines.

### Histology and quantification of cell size and cell number parameters in the fruit

In breaker stage, tomatoes were halved, and fruit diameter and pericarp thickness were measured at different points using a digital calliper. Pericarp samples were fixed in FAE (10% formaldehyde, 5% acetic acid, 50% ethanol) for 16 h, dehydrated and embedded in paraffin wax (Paraplast Plus)^60^. Then 7-μm sections were obtained using a Microm HM 330 microtome, mounted onto poly-l-lysine-coated slides and stained with 0.02% toluidine blue. The micrographs were captured by a Nikon Eclipse E600 microscope. Sections were used to estimate the number of cell layers and mean cell size that makes up the wild type and transgenic fruit pericarp. Measurements and counting were performed with the ImageJ software (NIH. https://imagej.nih.gov/ij). A total of four measurements per fruit were taken on a minimum of ten fruits per genotype. Cell wall fraction extraction was performed as described by Miedes and Lorences^65^.

Tomato pericarp nuclei were prepared as described in Serrani et al.^35^ and sorted by flow cytometry using a CyFlow cytometer (Partec) at the DNA Sequencing Service of the IBMCP in Valencia, Spain.

### Determination of fruit quality

Four representative fruits were collected from each plant in the mature-red stage as described in Renau-Morata^31^. Taste components were determined by capillary electrophoresis, as described by Cebolla-Cornejo et al.^61^. Sugars, fructose, glucose and sucrose, and organic acids, malic, citric, glutamic, glutamine and γ-amino butyric acid (GABA), were quantified. The derived sucrose equivalents, sucrose equivalents/citric acid and sucrose equivalents/malic acid ratios were calculated. Starch content was determined as described in Nebauer et al.^62^.

### Hormone determinations

Hormones (indole-3-acetic acid, and GA_1_ and GA_4_ gibberellins) were analysed in the fruits (17 days after anthesis (DAA) immature green fruits; approx. 2 cm diameter) by liquid chromatography-electrospray ionisation-tandem mass spectrometry (LC–ESI–MS/MS) using a Q-Exactive spectrometer (Orbitrap detector; ThermoFisher Scientific) by the Plant Hormone Quantification Service, IBMCP, Valencia, Spain. Four independent extractions were performed for each genotype. The fruit pericarp of three different plants (2 fruits per plant) were pooled in each biological replicate.

### Enzyme assays

Four hundred milligrams of frozen powder of young fruits (17 DDA immature green fruits) were re-suspended at 4 °C in 1.2 mL of 100 mM HEPES (pH 7), 2 mM EDTA and 5 mM dithiothreitol. The suspension was desalted (IVSS Vivaspin 500, Sartorius Biolab, Goettingen, Germany) following the manufacturer’s instructions and assayed for enzymatic activity. The sucrose synthase (SuSy, EC 2.4.1.13), acid invertase (INV, EC 3.2.1.26), ADP-glucose pyrophosphorylase (AGPase, EC 2.7.7.27) and UDP-glucose pyrophosphorylase (UGPase, EC 2.7.7.9) activities were assayed as described by Nebauer et al.^62^. SuSy and INV activities were measured in the sucrose breakdown direction. All the enzymatic reactions were performed at 37 °C. One unit (U) is defined as the amount of enzyme that catalyses the production of 1 μmol of product per min. Sampling was performed as indicated in the hormone analysis section.

### Gene expression analyses

Total RNA was extracted using RNeasy Plant Mini Kit (Qiagen, Hilden, Germany). The gene expression levels in the transgenic tomato plants were determined by qRT-PCR, following the procedures described in Corrales et al*.*^30^. The primer pairs used for amplification are described in Supplementary Table S3. The UBIQUITIN3 gene from *S. lycopersicum* was used as the reference gene^63^. The relative transcript levels of the genes were calculated by the 2^−ΔΔCT^ method^64^. For each gene, values are normalized to the mRNA levels in MM. Sampling was performed as indicated in the hormone analysis section.

### Statistical analyses

Data were analysed by a one-way ANOVA using the Statgraphics software (Statgraphics Centurion XVI, Statpoint Tech, Inc., Warrenton, VA, USA). The mean treatment values were compared (P < 0.05) by Fisher's least significant difference (LSD) procedure.

## Supplementary information

Supplementary file1.

## Data Availability

All supporting data can be found within the manuscript and its additional files.
