# Supplementary material for: The targeted overexpression of SlCDF4 in the fruit enhances tomato size and yield involving gibberellin signalling
Source: Sci Rep. 2020 Jun 30;10:10645. doi: 10.1038/s41598-020-67537-x (PMC7326986; doi:10.1038/s41598-020-67537-x)
Supplement: Supplementary file 1 — Supplementary file1. [file 41598_2020_67537_MOESM1_ESM.pdf]

# The targeted overexpression of *SlCDF4* in the fruit enhances tomato size and yield involving gibberellin signalling

Begoña Renau-Morata[1+], Laura Carrillo[3+], Jaime Cebolla-Cornejo[2], Rosa Victoria Molina[1], Raúl Martí[2], José Domínguez-Figueroa[3], Jesús Vicente-Carbajosa[3], Joaquín Medina[3*]; Sergio G. Nebauer[1*]

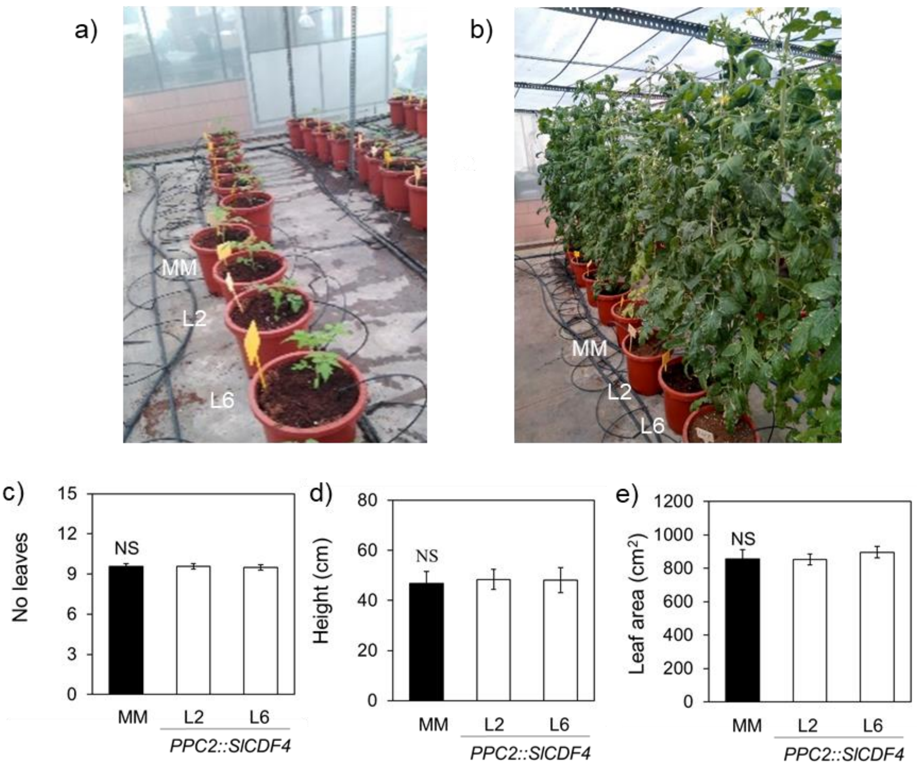


**Figure S1**. Phenotypic characterization of vegetative growth in *PP2C:SlCDF4* tomato plants. Representative plants at 4-5 leaf (a) and adult (b) growth stages of L2 and L6 transgenic lines. MM plants were used as controls. Number of leaves (c) and plant height (d) during the vegetative stage of growth (2-months-old plants). Area of the 5$^{th}$ mature leaf from the bottom (e), measured at the reproductive stage of the plant. Data are mean (±s.e.m.) of 10 different plants. Different letters indicate significant differences (LSD test; P<0.05). NS: not significant.

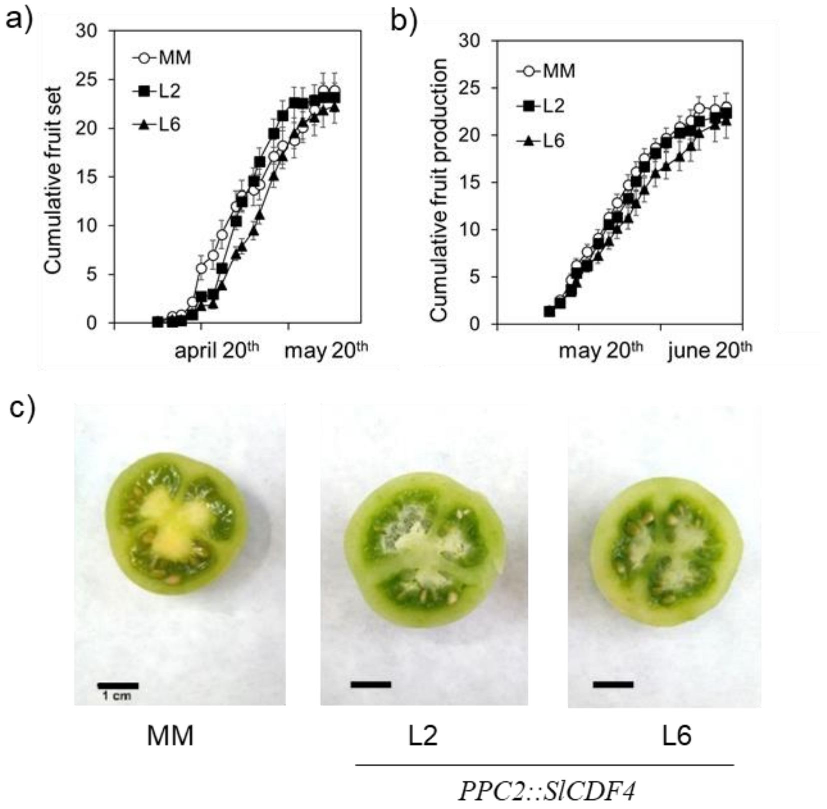


**Figure S2**. Characterization of fructification in *PP2C:SlCDF4* tomato plants. Cumulative fruit set (a) and fruits harvested at mature red stage (b) in L2 and L6 transgenic lines. Examples of equatorial section of 25 DAA fruits (c). MM plants were used as controls. Data are mean (±s.e.m.) of 10 different plants. Bars = 1 cm

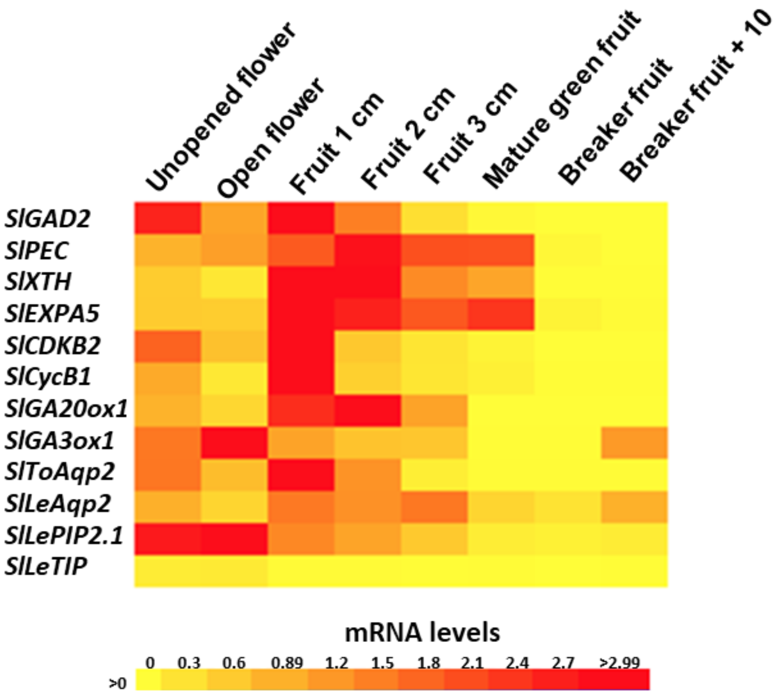


**Figure S3.** Heat map representation of mRNA expression levels of marker genes involved in cell cycle control, cell expansion, and hormone biosynthesis in different stages of tomato development. Data were obtained from BAR (http://bar.utoronto.ca/) using available data of genes involved in fruit development: *SlGAD2*, *SlPEC*, *SlXTH, SlEXP5*, *SlCDKB2*, *SlCycB1*, *SlGA20ox1*, *SlGA3ox1*, *SlToAqp2*, *SlLeAqp2*, *SlLePIP2.1* and *SlLeTIP* from the following tomato organs: unopened flower bud, opened flower, 1 cm fruit, 2 cm fruit, 3 cm fruit, mature green fruit, breaker fruit and breaker fruit + 10. The heat map was perfomed using BAR Heat Mapper software. The mRNA levels are presented using fold-change values compared to control. The colour scale representing the relative mRNA expression values is shown at the bottom. Genes highly expressed in the tissues are colored in red. *SlGAD2: glutamate decarboxylase*, *SlPEC: pectate lyase, SlEXP5: expansin, SlXTH: xyloglucan endotransglycoxylase-hydrolase, SlCDKB2: , SlCycB1: , SlGA20ox1: GA20 oxidase, SlGA3ox1: GA3 oxidase, SlToAqp2 , SlLeAqp2 and SlLePIP2: plasmatic membrane aquaporins and SlLeTIP: tonoplast aquaporin.*

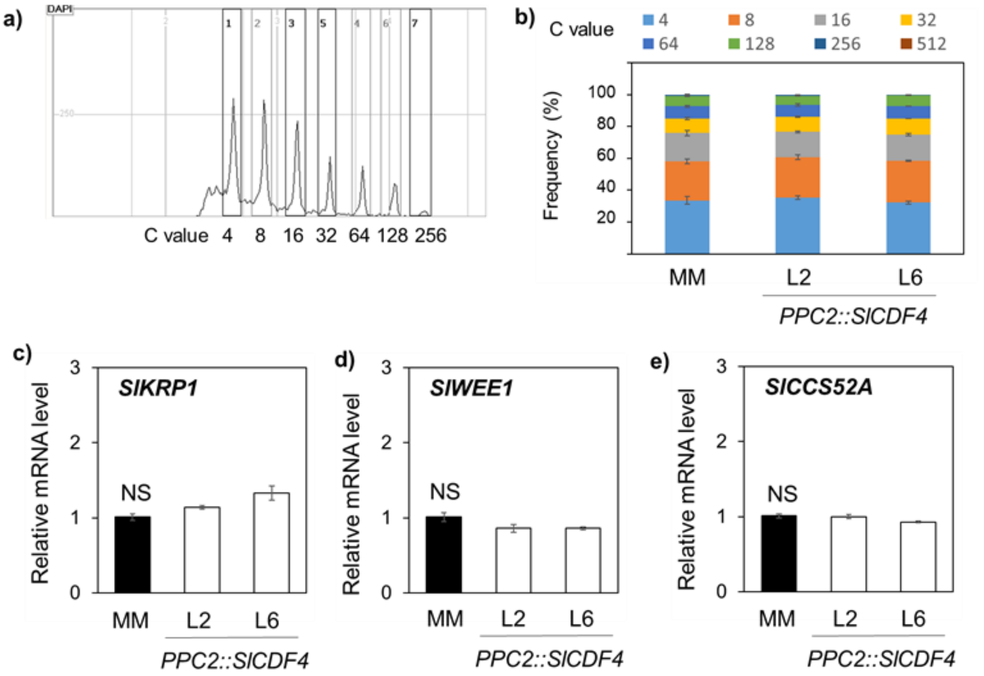


**Figure S4.** Ploidy levels in tomato pericarp of *PPC2:SlCDF4* plants. (a) Representative cytometry histogram of tomato pericarp at the breaker stage of *PPC2::SlCDF4* line L2. (b) Frequency (percentage values) of each ploidy level. Data are mean of 10 determinations in different fruits at breaker stage. The mRNA levels of the cyclin-dependent kinase inhibitor *SlKRP1* (c), the cell cycle-associated protein kinase *SlWEE1* (d), and the anaphase-promoting complex activator *SlCCS52A* (e) genes, related to endocycle control, were determined in fruits in the expansion phase (17 DAA). Data are the mean (±s.e.m.) of three biological replicates. Non transformed plants (MM) were used as controls. Transcript levels were normalized to the values of MM. Different letters indicate significant differences (LSD test; P<0.05). NS: not significant. The Partec FloMax software for Windows (www.sysmex.co.jp; Partec, Sysmex Corporation, Japan) was used to generate the flow cytometry histograms.

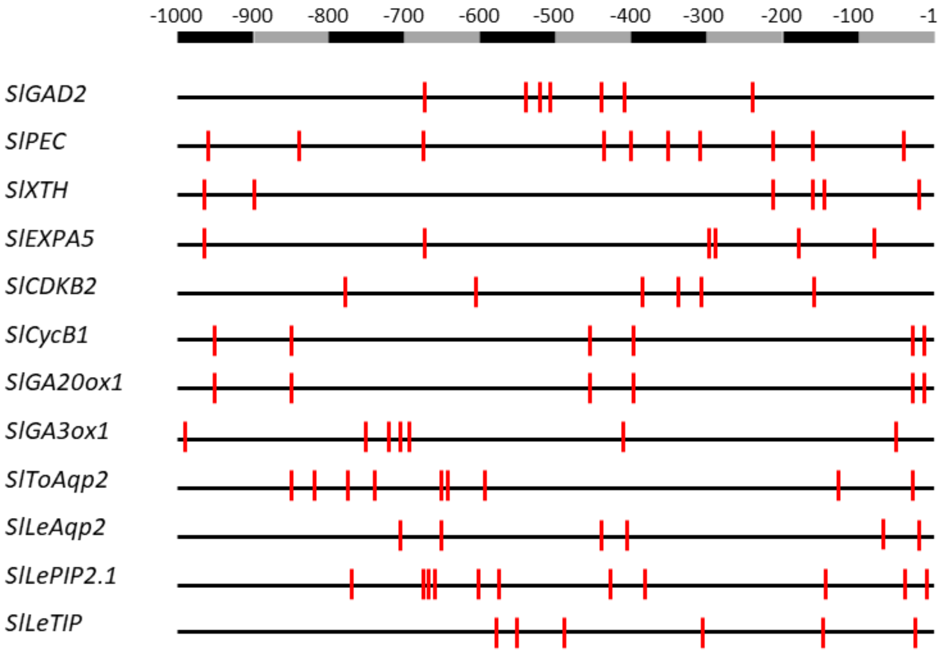


**Figure S5.** Promoter analysis for the presence of DOF-binding elements. The 1kb sequence upstream of the transcription initiation site of *glutamate decarboxylase* (*SlGAD2*), *pectate lyase* (*SlPEC), xyloglucan endotransglycosilase* (*SlXTH*), *expansin* (*SlEXPA5), B2-type cyclin dependent kinase* (*SlCDKB2*), *cyclin B1* (*SlCycB1*), *GA20 oxidase* (*SlGA20ox1*), *GA3 oxidase* (*SlGA3ox1*), *flavin monooxygenase* (*ToFZY*), *plasmatic membrane aquaporin* (*SlPIP1.2*), *plasmatic membrane aquaporin* (*SlPIP2.1*) and *tonoplast aquaporin* (*SlTIP2.3*) were analyzed. The resultant cis-regulatory elements (AAAG) are marked in red in the figure.

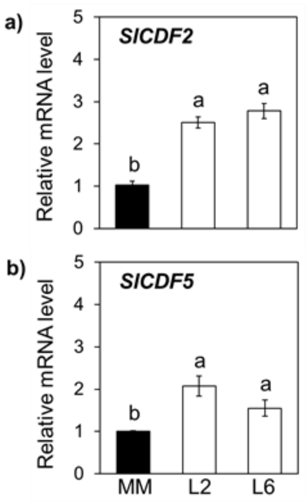


**Figure S6.** *SlCDF2* and *SlCDF5* transcript levels in *PPC2:SlCDF4* tomatoes. The mRNA levels of *SlCDF2* (a) and *SlCDF5* (b) genes were determined in fruits at the expansion phase (17 DAA). Transcript levels were normalized to the values of MM. Data are the mean (±s.e.m.) of four biological replicates. Non-transformed plants (MM) were used as controls. Different letters indicate significant differences (LSD test; $P<0.05$).

**Table S1.** Determination of parameters related to fruit size in *PPC2::SlCDF4* fruits at breaker stage. Fruit equatorial diameter, pericarp thickness, number of cell layers in the pericarp, mean area of the mesocarp cells and cell wall content are displayed. Mean seed weight and germination rates were also included. Non-transformed Moneymaker plants were used as controls. Data represent the means ± s.e.m. of different fruits (n=20)

| Line | Fruit diameter (cm) | Pericarp thickness (mm) | Pericarp no cell layers | Mesocarp cell mean area ($mm^2$) | Cell wall content (mg/100 mg DW) | Seed weight (g/1000 seeds) | Germination rate (%) |
|---|---|---|---|---|---|---|---|
| MM | 4.2 ± 0.2 b | 2.9 ± 0.1 b | 13.3 ± 0.5 b | 0.07 ± 0.01 c | 49.8 ± 0.5 b | 3.21 ± 0.10 | 93 |
| *PPC2::SlCDF4* L2 | 7.3 ± 0.3 a | 5.3 ± 0.3 a | 15.1 ± 0.4a | 0.13 ± 0.00 a | 53.5 ± 0.6 a | 3.12 ± 0.23 | 91 |
| *PPC2::SlCDF4* L6 | 6.8 ± 0.4 a | 5.3 ± 0.1 a | 15.9 ± 0.5 a | 0.09 ± 0.01 b | 54.8 ± 0.8 a | 2.98 ± 0.37 NS | 95 NS |

For each parameter, different letters indicate significant differences (P<0.05). NS: not significant.

**Table S2.** Effect of the overexpression of *SlCDF4* in fruit (lines L2 and L6) on compounds related to organoleptic quality in ripe tomato fruits. Fruits of non-transformed Moneymaker (MM) plants were used as controls. DM: dry matter; SSC: soluble sugar content (ºBrix); MA: Malic acid; CA: Citric acid; GABA: γ-amino butyric acid; SE/CE: sucrose equivalent to citric acid ratio; CA/FW: fresh weight. Starch content is also displayed. Values are mean of four different determinations (±s.e.m.) in different fruits at the mature red stage.

| Genotype | Average DM per fruit (g) | SSC (ºBrix) | Malic acid (mg/g FW) | Citric acid (mg/g FW) | Glutamic acid (mg/g FW) | GABA (mg/g FW) | Glutamine (mg/g FW) | Fructose (mg/g FW) | Glucose (mg/g FW) | SE/CE | MA/CA | Starch (μg/mg DW) |
|---|---|---|---|---|---|---|---|---|---|---|---|---|
| MM | 1.1±0.0 | 5.7±0.2 | 1.7±0.1 | 8.3±0.4 | 2.9±0.2 | 0.74±0.03 | 1.4 ± 0.1 | 18.4±0.4 | 15.5±0.4 | 4.3±0.1 | 0.21±0.01 | 31±2 |
| *PPC2::SlCDF4* | | | | | | | | | | | | |
| L2 | 2.2±0.1* | 4.6±0.1* | 2.4±0.1* | 4.3±0.1* | 3.8±0.5 | 1.51±0.04* | 2.8 ± 0.6* | 13.5±1.4* | 10.8±1.1* | 5.0±0.3* | 0.55±0.02* | 34±4 |
| L6 | 1.7±0.1* | 4.4±0.2* | 2.3±0.2* | 3.8±0.2* | 2.5±0.4 | 1.15±0.23 | 1.9 ± 0.8 | 15.1±0.6 | 12.0±0.3* | 5.4±0.2* | 0.62±0.06* | 32±1 |

* Indicates significant differences compared to the MM (Dunnett test, 95% confidence)

**Table S3.** Primers used in RT-qPCR analyses

| Gene | | Gene ID | Primer sequence |
|---|---|---|---|
| *Glutamate Decarboxylase* | *SlGAD2* | Solyc07g043420.2 | CGTCGTTGTACCACCACTACGC |
| | | | ACGCGAAAGTCGAGTGAACGG |
| *Protein kinase WEE1* | *SlWEE1* | Solyc09g074830.2 | CAAATTTGAAATCTCCCCAA |
| | | | TGGAAATGGATCTTCTTGTA |
| *Anaphase Promoting Complex activator CCS52A* | *SlCCS52A* | Solyc08g080080.2 | AAACCCCATCAAGAACAAAT |
| | | | AAGATCTACTCGGAATGAAA |
| *Cyclin-dependent kinase inhibitor* | *SlKRP1* | Solyc09g091780.2 | GGAGAGCACACCTTGCAGTTT |
| | | | TACTCTGCCGTTGGCCTCAT |
| *Auxin/indole acetic acid (Aux/IAA) protein* | *SlIAA17* | Solyc06g008590.2 | CAAGAATTATTTGATGCCTTAACCAA |
| | | | ACTATTCAAAAGGTCCATCAGTTTCC |
| *Phosphoglucomutase* | *SlPGM* | Solyc04g045340.2 | TAGTTGCACGTATGGGATTGTC |
| | | | GGCAACACCTTTCAAACCT |
| *Pectate lyase* | *SlPEC* | Solyc06g083580.2 | ATGGGAAGGATCATGGAGACAGTGG |
| | | | AAGGAAGAGGACTTCGCAGCTAAGC |
| *Xyloglucan endotransglycosilase* | *SlXTH* | Solyc03g031800.2 | CTGCCACGCCACAAGAAGTCC |
| | | | TTTGACGAACCCAACGAAGTCTCC |
| *Expansin* | *SlEXPA5* | Solyc02g088100.2 | AAGGGTTCAAGAACTCAATGGCAAC |
| | | | ACCATCGCCTGTAGTGACCTTAAAG |
| *B2-type cyclin dependent kinase* | *SlCDKB2* | Solyc04g082840.2 | ATGCTGGTAAGAGTGTATCGG |
| | | | CGGAGAGTAGTTGGAGGAAC |
| *Cyclin B1* | SlCycB1 | Solyc10g078330.1 | CGTTACTAGGAGGTCTGCTG |
| | | | CCTTTAGTTACAAGAGGCTTCG |
| *GA20 oxidase* | *SlGA20ox1* | Solyc03g006880.2 | CTCATTTCTAATGCTCATCGTTATA |
| | | | AGATGATTCTTTCTTAGCGGAG |
| *GA3 oxidase* | *SlGA3ox1* | Solyc06g066820.2 | GGCATTAGTAGTTAATATAGGTGA |
| | | | AAATAAGCTACAGAAAGTCGATATC |

| | | | |
|---|---|---|---|
| *Flavin containing monooxygenase* | ToFZY | Solyc06g065630.2 | CAATGTGCCTTCTTGGTTTAAG |
| | | | GCATCATTTGCAGTCCCTAATAT |
| *Plasmatic membrane aquoporin* | *LeAqp2* | Solyc01g094690.2 | GTTCCAGCCATCTTTGTTTGG |
| | | | TGTAACCATGGGCAACAACATT |
| *Plasmatic membrane aquoporin* | *LePIP2.1* | Solyc10g055630.1 | CATCATCATGGAAAGGACTATGTTG |
| | | | AAGCTCAGCCATGTCGAGAAG |
| *Tonoplast aquoporin* | *LeTIP* | Solyc06g060760.2 | GGAACCATTGCACCCATTG |
| | | | GGCCAGCTGCCAAAATGT |
